# Editorial: Self-Eating on Demand: Autophagy in Cancer and Cancer Therapy

**DOI:** 10.3389/fonc.2017.00302

**Published:** 2017-12-07

**Authors:** Jon D. Lane, Patrizia Agostinis

**Affiliations:** ^1^Cell Biology Laboratories, School of Biochemistry, University of Bristol, Bristol, United Kingdom; ^2^Cell Death Research and Therapy (CDRT) Lab, Department of Cellular Molecular Medicine, KU Leuven University of Leuven, Leuven, Belgium

**Keywords:** calcium signaling, tumor microenvironment, secretion, microRNAs, hypoxia, melanoma, pancreatic cancer, immunosurveillance

**Editorial on the Research Topic**

**Self-Eating on Demand: Autophagy in Cancer and Cancer Therapy**

The field of autophagy has grown enormously over the past 10–15 years, with rapid advances in our understanding of the regulatory mechanisms that control autophagy pathways in mammalian systems, and an improved understanding of the physiological influences of autophagy in health and disease. Supporting such progress, there has been substantial diversification in assessment and modulation tools (1), assisted by the advancement of model reporter systems. Indeed, we are now starting to realize the potential for autophagy control for novel practical applications, including disease intervention and biotechnology. With the aim of promoting, supporting, and streamlining cooperative European research networks to realize the enormous potential of autophagy in the clinic and in industry, a collaborative consortium—called TransAutophagy—was approved in November 2015 in the framework of the Horizon 2020 Program as a European Union CO-operation in Science and Technology (COST) Action (CA15138^1^) (2). Sponsored for 4 years, this network includes more than 250 scientists from 21 countries, with each participant actively engaged in basic and/or translational autophagy research.

TransAutophagy comprises five different thematic Working Groups with activities designed to synergize and support translation of our ever-advancing basic autophagy knowledge into biomedical and biotechnological applications (2). Targeting the complex physiological and metabolic changes inherent within cancer cells during transformation, tumor growth, and metastasis, through manipulation of autophagy regulatory networks, is a key objective because emerging evidence indicates that autophagy capability underpins a cancer cell’s ability to face the increasingly hostile tumor microenvironment. Here, poor nutrient availability and elevated cellular stress place demands upon the cancer cell for an increased capability to adapt and survive. Several lines of evidence have established that cancer cells use autophagy as a highly plastic and dynamic mechanism to either repress initial steps in carcinogenesis or to support the survival and growth of established tumors (3). Moreover, it is becoming increasingly clear that autophagy regulates the intersection between cancer and stromal cells in tumors. The tumor-suppressing role of autophagy involves, e.g., (i) maintenance of genetic/genomic stability; (ii) preservation of bioenergetics; (iii) reduction and control of (mutagenic/damaging) reactive oxygen species; (iv) degradation of oncogenic proteins; (v) activation of tumor-suppressing mechanisms like oncogene-induced senescence and autophagic cell death; (vi) reduction of chronic inflammation; and (vii) regulation of immunosurveillance mechanisms [reviewed in Ref. (3, 4)]. This collection of reviews—comprising this research topic—addresses emerging traits highlighting how autophagy shapes the cancer cell-tumor microenvironment crosstalk.

The review of Mathiassen et al. (Cecconi’s lab) discusses mounting evidence for new regulatory intersections between autophagy and the cell cycle, which need to be urgently validated *in vivo*. At the mechanistic level, the tumor suppressor role of autophagy has been ascribed to its vital cell-autonomous functions in mitigating damage and maintaining cellular integrity during metabolic stress. An emerging and intriguing link, which is discussed in the review of Kania et al. (Bultynck’s/Parys’s labs), is the regulation of autophagy in cancer cells through Ca^2+^ transfer from the ER to mitochondria *via* the inositol 1,4,5-trisphosphate receptor (IP3R) at ER–mitochondria contact sites. In a developing research area with enormous potential, the impact of miRNA-mediated autophagy regulation on the tumor microenvironment and cancer growth, and their potential as cancer biomarkers and therapeutic targets, is discussed in the review of Gozuacik et al. (Gozuacik’s lab).

In established tumors, elevated levels of autophagy are often associated with poorly oxygenated regions where the demand for nutrients and the need to withstand diverse metabolic stresses are increased. As further discussed in the review of Viry et al. (Janji’s lab), cancer cell-associated autophagy in hypoxic tumors plays a crucial role in modulating immunosurveillance and in fostering the immunosuppressive tumor microenvironment, by suppressing key mechanisms of innate and adaptive antitumor immunity, thus favoring tumor outgrow and dissemination. Consistent with this pro-tumorigenic role, advanced tumors often display an “autophagy-lysosomal addiction,” which appears to be required to maintain their energy balance through the recycling of intracellular components into biosynthetic pathways or ATP synthesis and to regulate secretion of pro-tumorigenic factors. In the review of New et al. (Tooze’s lab), the idea that advanced and aggressive mutant *KRAS*-driven tumors (such as pancreatic ductal adenocarcinomas) exploit a heightened autophagy–lysosomal pathway under the transcriptional control of the MiF/TFE factors to support energy metabolism and to allow growth under conditions of energy deficit and metabolic stress is discussed (5). Furthermore, the review of Iovanna (Iovanna’s lab) highlights the key role played by the pancreatitis-associated vacuolar protein 1 (VMP1) in pancreatic acinar cells and how its elevated expression drives early autophagy and cooperates with the *KRAS* oncogene to promote carcinogenesis in the pancreas.

Another emerging aspect linking autophagy to tumor progression, discussed in the review of Keulers et al. (Rouschop’s lab), is the ability of advanced cancer cells to use autophagy as a trafficking and export mechanism of pro-tumorigenic factors, such as pro-inflammatory/pro-angiogenic cytokines or chemotactic/pro-invasive molecules. This cancer cell-autonomous trait further illustrates the plasticity of tumor-associated autophagy, which can enable and modulate the crosstalk between cancer and stromal cells thereby affecting the tumor microenvironment, a property that needs to be taken into consideration when considering therapeutic approaches. Based on the growing relevance of tumor-associated autophagy, many labs are developing and testing the effects of autophagy modulators in cancer therapy. The recognition of the prevalent—albeit not unique—cytoprotective and stress adaptation roles of autophagy in advanced cancers has led to the assumption—as supported by *in vitro* and preclinical data—that blocking cancer cell-intrinsic autophagy may curtail cancer cell resistance to chemotherapy, thereby improving therapy outcome. Thus, the first-generation autophagy blockers, e.g., chloroquine and its derivative hydroxychloroquine (6, 7) are currently being tested in different clinical trials to potentiate patients’ responses to a variety of anticancer regimens.^2^ On the other hand, as autophagy can control both cell death and survival programs, the induction of autophagic cancer cell death elicited by certain anticancer therapies, may offer a therapeutically attractive strategy, especially when cancer cells display resistance to apoptosis, as discussed in the review by Fulda (Fulda’s lab). Finally, although autophagy is a highly dynamic process, the expression of certain autophagy genes in aggressive tumors like melanoma, may provide novel independent prognostic biomarkers for early stage neoplasms, as discussed in the review of Tang et al. (Lovat’s lab). This may help to identify patients at risk of disease progression, thus facilitating earlier patient therapeutic intervention and stratification for personalized therapeutic approaches.

## Author Contributions

Authors contributed equally.

## Conflict of Interest Statement

The authors declare that the research was conducted in the absence of any commercial or financial relationships that could be construed as a potential conflict of interest.

## Footnotes

^1^http://cost-transautophagy.eu.

^2^https://clinicaltrials.gov/.
